# Preoperative sarcopenia and its impact on postoperative complications in laparoscopic anti-reflux surgery: a clinical analysis

**DOI:** 10.3389/fsurg.2026.1723324

**Published:** 2026-03-11

**Authors:** Zhong-Yu Wang, Yu Liu, Jie Lin, Fan-Ke Wang, Hong-Fei Pang, Yu-Hang Liu, Ming Wei, Yuan-Yuan Wang

**Affiliations:** 1 Gastrointestinal Disease Center, The First Hospital of Hebei Medical University, Shijiazhuang, Hebei, China; 2 Department of Breast Surgery, Hengshui People’s Hospital, Hengshui, Hebei, China

**Keywords:** anti-reflux surgery, complication, health, prognosis, sarcopenia

## Abstract

**Background:**

To investigate the influence of sarcopenia on postoperative outcomes in gastroesophageal reflux disease patients undergoing concomitant laparoscopic hiatal hernia repair and laparoscopic fundoplication.

**Methods:**

Retrospective analysis was conducted on 69 patients who underwent laparoscopic hiatal hernia repair combined with laparoscopic fundoplication in the Department of Gastroenterology at the First Hospital of Hebei Medical University from September 2024 to May 2025. Skeletal muscle area at the L3 level was measured using abdominal CT scans within 10 days preoperatively to diagnose sarcopenia. Patients were divided into sarcopenia and non-sarcopenia groups. General clinical data, laboratory findings and postoperative complications were compared between the two groups to investigate the relationship between sarcopenia and postoperative complications following laparoscopic hiatal hernia repair combined with laparoscopic fundoplication.

**Results:**

Patients in the sarcopenia group were significantly older and exhibited lower BMI, lower preoperative scores on the gastroesophageal reflux disease questionnaire, and reduced acid exposure time percentages (all *P* < 0.05). Furthermore, this group demonstrated significantly lower postoperative serum albumin levels and prolonged gastrointestinal function recovery time (*P* < 0.05). Multivariate linear regression analysis revealed that, after adjustment for potential confounders including gender, age, and neutrophil percentage, factors such as gender, age, presence of comorbid pulmonary disease, BMI, and preoperative albumin level were independently associated with postoperative albumin levels (all *P* < 0.05). Additionally, multivariate logistic regression identified preoperative sarcopenia and advanced age as independent risk factors for delayed recovery of gastrointestinal function following laparoscopic hiatal hernia repair combined with fundoplication (*P* < 0.05).

**Conclusion:**

​Our findings clearly indicate that while preoperative sarcopenia does not elevate the risk of postoperative dysphagia, it significantly delays the recovery of gastrointestinal function and leads to lower albumin levels following surgery in gastroesophageal reflux disease patients. This underscores the critical clinical importance of recognizing sarcopenia as a modifiable preoperative risk factor. We therefore propose the integration of routine sarcopenia screening into the preoperative assessment for gastroesophageal reflux disease patients. For those diagnosed with sarcopenia, a targeted prehabilitation protocol emphasizing nutritional support and physical training could be implemented to potentially enhance surgical tolerance and recovery outcomes. Future studies are warranted to validate the efficacy of such prehabilitation strategies and to further investigate the precise molecular mechanisms through which sarcopenia impedes postoperative recovery.

## Introduction

Laparoscopic hiatal hernia repair (LRHH) combined with laparoscopic fundoplication (LF) is a standard surgical approach for treating hiatal hernia (HH) complicated by gastroesophageal reflux disease (GERD) (1). Its primary objectives include alleviating reflux symptoms, improving quality of life, and achieving withdrawal from pharmacotherapy (2). In recent years, despite advances in surgical techniques, postoperative complications such as hypoalbuminemia, delayed gastrointestinal function recovery, and dysphagia (3) continue to impair patient recovery. The occurrence of complications after anti-reflux surgery is related to multiple factors, such as age (4), gender (5), and weight (6).

Sarcopenia, initially described by Rosenberg et al., is an age-related condition characterized by progressive loss of skeletal muscle mass, strength, and physical performance (7–9). As an indicator of systemic malnutrition, it has gained increasing clinical attention (10). Manifesting as generalized skeletal muscle depletion and functional decline, sarcopenia is recognized as a potential risk factor for surgical trauma and has been demonstrated to correlate with abdominal surgery complications (11). And with the aging of the population, sarcopenia has become a significant public health problem affecting the health of the older adults. Evaluating its impact on outcomes in common surgeries is crucial for optimizing public health resources and formulating perioperative management strategies. However, its relevance to laparoscopic fundoplication remains understudied.

This retrospective study analyzed the incidence of postoperative complications in sarcopenic vs. non-sarcopenic patients undergoing combined LRHH and LF at the Department of Gastroenterology, First Hospital of Hebei Medical University, between September 2024 and May 2025.

## Methods

### Patients and grouping

This study conducted a retrospective analysis of clinical data from patients who underwent concomitant LRHH and LF at the Department of Gastroenterology, the First Hospital of Hebei Medical University, from September 2024 to May 2025. Patient selection criteria were applied as follows: Inclusion required (1) a diagnosis of HH complicated by GERD was established and a repair of the hiatal hernia combined with fundoplication was successfully performed (2) availability of complete preoperative clinical documentation. Exclusion criteria comprised (1) suboptimal abdominal CT image quality and (2) presence of confounding conditions potentially inducing muscle weakness, including sequelae of cerebrovascular accidents, spinal cord disorders, malignant neoplasms, or consumptive diseases. Following screening, 69 eligible cases were stratified into sarcopenia (*n* = 21) and non-sarcopenia (*n* = 48) groups based on preoperative L3 skeletal muscle index assessment.

### Diagnostic criteria for sarcopenia

**Figure 1:**
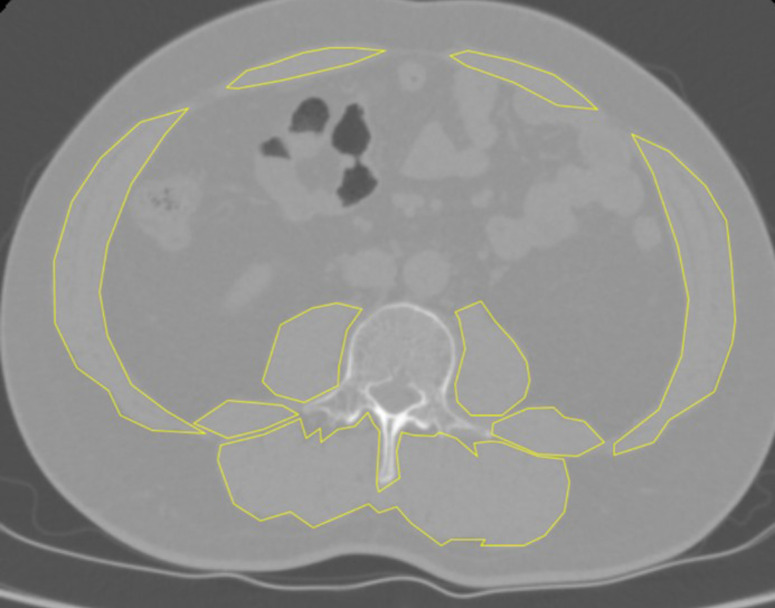
Schematic illustration of skeletal muscle measurement boundaries at the L3 vertebral level.

Computed tomography (CT) is currently regarded as a reference standard for skeletal muscle mass quantification and is widely employed for sarcopenia diagnosis across diverse clinical populations (12–14). Evidence confirms that measurements at the third lumbar vertebra level accurately reflect whole-body muscle and adipose composition while maintaining prognostic validity in both normal-weight and overweight individuals (15). In this study, all participants underwent non-contrast abdominal CT within 10 days preoperatively. Skeletal muscle contours at the L3 level were manually delineated using Image J software by two independent radiologists who were blinded to the patient groups and clinical outcomes, encompassing the rectus abdominis, abdominal wall musculature, psoas major, quadratus lumborum, and erector spinae (Figure 1). The inter-observer reliability was assessed using the intraclass correlation coefficient (ICC), which demonstrated excellent agreement (ICC = 0.9). Any discrepancies in measurements were resolved through consensus. Sarcopenia diagnosis adhered to Asian Working Group for Sarcopenia criteria (16), with the skeletal muscle index (SMI) calculated as the total L3 cross-sectional area (cm²) divided by height squared (m²). Gender-specific thresholds defined sarcopenia as SMI <36.2 cm²/m² for males and <29.6 cm²/m² for females (17).

### Observe the metrics

The following variables were documented in both cohorts: ​baseline characteristics​ including gender, age, body mass index (BMI), leukocyte count, neutrophil percentage, hemoglobin, albumin, comorbidities, American Society of Anesthesiologists (ASA) classification, gastroesophageal reflux disease questionnaire (GERDQ) score, acid exposure time (AET), and DeMeester score; ​postoperative complications​ comprising hypoalbuminemia (serum albumin <30 g/L), severe dysphagia (defined as inability to tolerate solid foods beyond postoperative day 7, requiring endoscopic intervention or nutritional support), and delayed gastrointestinal function recovery (defined as time to first flatus >72 h, based on prior studies indicating that prolonged ileus beyond this threshold is clinically significant in abdominal surgery (18); and ​supplementary outcomes: operative duration (minutes), intraoperative blood loss (mL), and hospital length of stay.

### Preoperative preparation

Preoperative interventions involved cessation of smoking and alcohol consumption, along with cardiopulmonary exercise training. Patients were instructed to fast from solid foods for at least 6 h and clear liquids for 2 h prior to surgery. Standard preoperative diagnostic workup included thoracic and abdominal computed tomography, barium upper gastrointestinal series, and 24-hour ambulatory esophageal pH monitoring to facilitate surgical risk stratification.

### Surgical modality

All 69 patients underwent standardized LRHH with LF performed by our dedicated surgical team. Following general endotracheal anesthesia and five-port access, the procedure comprised seven critical phases: (1) Liver retraction and division of the gastrohepatic ligament; (2) Complete dissection of the right diaphragmatic crus and esophageal anterior peritoneal layer; (3) Mobilization of gastrophrenic and esophagophrenic ligaments with exposure of the left crus; (4) Esophageal mobilization to achieve ≥5 cm intra-abdominal length; (5) Hernia sac reduction followed by interrupted crural approximation using 3-0 silk sutures, with mesh reinforcement for hiatal defects >5 cm; (6) Gastric fundus traction assessment to determine optimal fundoplication technique; and (7) Tension-free wrap construction encircling the distal esophagus with 2-0 silk sutures, as standardized in the *Seven-Phase Laparoscopic Hiatal Hernia Repair Protocol* (19).

### Postoperative management

Standard postoperative care included intravenous anti-inflammatory agents, proton pump inhibitors (PPIs) for acid suppression, and fluid-electrolyte replacement. Patients initiated oral intake with water sips on postoperative day (POD) 1, advancing to clear liquids upon confirmed bowel function recovery. Mandatory laboratory assessments on POD 1 encompassed complete blood count, Liver and kidney function tests, electrolytes, and C-reactive protein (CRP). Discharge eligibility required fulfillment of all criteria by POD 3-4.

### Statistical analysis

Statistical analyses were performed using SPSS version 27.0. Continuous variables underwent normality assessment via Shapiro–Wilk tests. Normally distributed data were expressed as mean ± standard deviation (x¯ ± SD) and compared using independent samples t-tests. Non-normally distributed variables were presented as median (interquartile range, IQR) with Mann–Whitney U tests for group comparisons. Categorical variables were summarized as frequencies (%) and analyzed with Pearson's *χ*² tests. Variables with a *P*-value < 0.05 in univariate analysis, along with clinically relevant factors considered important based on existing literature (e.g., age, gender), were selected as candidates for inclusion in the multivariate models. Multivariate linear regression identified predictors of continuous outcomes, while logistic regression determined risk factors for binary complications. Statistical significance was defined as two-tailed *P* < 0.05 for all analyses.

### Ethical approval

Ethical approval for this retrospective study was obtained from the Ethics Committee of the First Hospital of Hebei Medical University [Ethics Review (2025) No.174].

## Results

### General clinical data of the two groups

Patients in the sarcopenia group were significantly older (*P* = 0.004) and had lower preoperative BMI (*P* = 0.038), reduced GERDQ scores (particularly for reflux syndrome A), shorter acid reflux duration, and decreased longest reflux episode duration (all *P* < 0.05) compared to the non-sarcopenia group. Preoperative acid exposure time (AET) was also lower in the sarcopenia cohort (*P* = 0.041). No statistically significant intergroup differences (*P* > 0.05) were observed for gender, preoperative weight, comorbidities (hypertension, diabetes mellitus), pulmonary disorders, ASA classification, number of acid reflux episodes, DeMeester score, hemoglobin, white blood cell count, neutrophil percentage, serum albumin, triglycerides, cholesterol levels, or prevalence of Barrett's esophagus on preoperative endoscopy. Complete data are presented in Table 1. Fundoplication techniques included Nissen (*n* = 35), Toupet (*n* = 6), and Dor (*n* = 28), with no significant difference in distribution between sarcopenia and non-sarcopenia groups (*P* = 0.47).

**Table 1:** Comparison of the general data of the two groups.

Variable	Non-sarcopenia	Sarcopenia	*P* value
Gender			0.799
Male	29	12	
Female	19	9	
Age (years)	53.90 ± 11.89	63.81 ± 14.01	0.004
High blood pressure and/or diabetes mellitus			0.121
Yes	20	13	
No	28	8	
Lung disease			0.268
Yes	10	7	
No	38	14	
ASA grading			0.200
1∼2	24	7	
3∼4	24	14	
Weight (kg)	70.85 ± 11.67	65.45 ± 10.17	0.071
BMI (kg/m^2^)	25.78 ± 3.64	23.71 ± 3.97	0.038
A symptom	6 (4, 6)	4 (1, 6)	0.017
B symptom	6 (2.25, 6.00)	4 (1.50, 6.00)	0.536
C symptom	2 (0, 3)	2 (0, 3)	0.951
GERDQ	10.75 ± 2.85	8.71 ± 4.24	0.022
Acid reflux time (min)	196.95 (48.53, 292.79)	76.1 (17.15, 231.38)	0.043
AET (%)	13.95 (3.43, 19.97)	5.30 (1.20, 16.30)	0.041
Number of acid refluxes	35.50 (15.75, 60.75)	29.00 (13.00, 45.25)	0.370
Long regurgitation duration (min)	27.5 (7.40, 40.31)	13.2 (3.3, 29.5)	0.037
DEMEESTER score	30.630 (10.250, 75.843)	19.510 (7.685, 47.999)	0.334
Hb (g/L)	142.75 ± 19.502	137.67 ± 14.333	0.287
WBC (*10^9^/L)	6.165 ± 1.998	6.200 ± 2.190	0.948
Neutrophil percentage (%)	62.773 ± 10.8631	61.967 ± 16.7945	0.812
Platelet (*10^9^/L)	239.00 (206.50, 284.75)	211.00 (183.00, 252.50)	0.092
Serum albumin (g/L)	44.20 ± 4.14	43.67 ± 4.16	0.639
Triglycerides (mmol/L)	1.5531 (1.1375, 1.6875)	1.5531 (0.9600, 1.6000)	0.292
Cholesterol (mmol/L)	5.2984 ± 1.07261	5.1000 ± 0.81852	0.453
Barrett's Esophagus			0.542
Yes	1	1	
No	47	20	

### The intraoperative and postoperative conditions of the two groups were compared

Compared to the non-sarcopenia group, sarcopenic patients exhibited significantly ​lower postoperative serum albumin levels​ (*P* = 0.005) and ​prolonged gastrointestinal function recovery time​ (*P* = 0.007). The proportion of patients with hypoalbuminemia (albumin <30 g/L) was 43% in the sarcopenia group vs. 21% in the non-sarcopenia group. No significant intergroup differences (*P* > 0.05) were observed for operative duration, intraoperative blood loss, hernia classification, hiatal hernia area (20), postoperative hemoglobin, white blood cell count, neutrophil percentage, C-reactive protein levels, length of hospital stay, or rates of severe dysphagia. Comprehensive data are presented in Table 2.

**Table 2:** Comparison of intraoperative and postoperative conditions between the two groups.

Variable	Non-sarcopenia	Sarcopenia	*P* value
Postoperative Hb (g/L)	132.45 ± 17.534	126.19 ± 13.735	0.151
Postoperative WBC (*10^9^/L)	9.334 ± 2.4561	9.010 ± 2.1443	0.601
Postoperative neutrophil percentage (%)	79.974 ± 7.1144	80.243 ± 8.5795	0.893
Postoperative CRP (mg/L)	17.295 (9.5575, 28.7200)	11.900 (8.5800, 25.3777)	0.144
Postoperative serum albumin (g/L)	38.06 ± 3.17	35.72 ± 2.89	0.005
Duration of surgery (min)	123.83 ± 30.781	132.90 ± 50.646	0.363
Intraoperative blood loss (mL)	20 (10, 20)	20 (10, 20)	0.851
Intraoperative hernia classification			0.875
I	29	13	
II	1	0	
III	15	6	
IV	3	2	
Hiatal hernia area (cm^2^)	7.56 (6.04, 7.62)	7.56 (6.04, 9.12)	0.958
Length of postoperative hospital stay (day)	4 (3.00, 4.75)	5 (3.50, 7.00)	0.100
Time to recovery of gastrointestinal function after surgery (day)	1 (1, 3)	3 (1.5, 4)	0.007
Severe dysphagia			0.910
Yes	5	2	
No	43	19	

## Univariate and multivariate analyses of postoperative Alb levels and postoperative gastrointestinal function recovery time

### Postoperative serum albumin level

Univariate analysis identified significant associations between postoperative albumin levels and sarcopenia status, gender, age, pulmonary comorbidities, body weight, BMI, operative duration, and preoperative albumin (Table 3). Subsequent multivariate linear regression incorporating significant univariate predictors (*P* < 0.05) and clinically relevant variables revealed that ​pre-existing pulmonary disease, BMI, and preoperative albumin​ were independent risk factors for post-fundoplication hypoalbuminemia (all *P* < 0.05) (Table 4).

**Table 3:** Univariate analysis of albumin levels after surgery.

Variable	Postoperative serum albumin $\bar{x}$±SD/M (*P*_25_, *P*_75_)/*β* (95%CI)	*χ*2/t/Z value	*P* value
Sarcopenia or not		−2.890	0.005
Yes	35.719 ± 2.888		
No	38.056 ± 3.173		
Gender		2.282	0.026
Male	38.109 ± 3.399		
Female	36.326 ± 2.807		
Age (years)	−0.099 (−0.153, −0.044)	−3.605	<0.001
High blood pressure and/or diabetes mellitus		−0.692	0.492
Yes	37.061 ± 3.120		
No	37.605 ± 3.395		
Lung disease		−2.189	0.032
Yes	35.885 ± 3.604		
No	37.822 ± 3.016		
Weight (kg)	0.353 (0.035, 0.165)	3.087	0.003
BMI (kg/m^2^)	0.221 (0.021, 0.420)	2.209	0.031
Duration of surgery (min)	−0.267 (−0.043, −0.003)	−2.269	0.026
Preoperative serum albumin (g/L)	0.468 (0.199, 0.540)	4.336	<0.001

**Table 4:** Multivariate linear regression analysis affecting postoperative albumin levels.

Variable	Statistics or effect size of postoperative serum albumin	*P* value
Sarcopenia or not	0.132 (−0.592, 2.445)	0.227
Gender	−0.330 (−4.326, −0.012)	0.049
Age (years)	−0.184 (−0.107, 0.017)	<0.001
Lung disease	0.211 (0.109, 3.055)	0.036
Weight	−0.414 (−0.275, 0.039)	0.139
BMI (kg/m^2^)	0.601 (0.057, 0.962)	0.028
Duration of surgery (min)	−0.175 (−0.032, 0.002)	0.088
Preoperative serum albumin (g/L)	0.325 (0.069, 0.444)	0.008

### Time to recovery of gastrointestinal function after surgery

Univariate analysis demonstrated that ​sarcopenia status was the sole factor significantly associated with delayed gastrointestinal recovery​ (Table 5). To control for potential confounding, variables including gender, age, BMI, and preoperative albumin—despite not being statistically significant in univariate analysis—were included in the multivariate model due to their established clinical relevance to postoperative recovery.​ Multivariate regression confirmed that ​sarcopenia remained an independent risk factor for delayed gastrointestinal recovery​ after risk adjustment (Table 6).

**Table 5:** Univariate analysis affecting the prolongation of postoperative gastrointestinal function recovery time.

Variable	Prolonged recovery of gastrointestinal function (*n* = 17)	Normal (*n* = 52)	χ2/t/Z value	*P* value
Sarcopenia			8.587	0.003
Yes	10 (47.6)	11 (52.4)		
No	7 (14.6)	41 (85.4)		
Gender			0.003	0.954
Male	10 (24.4)	31 (75.6)		
Female	7 (25.0)	21 (75.0)		
Age (years)	54.24 ± 16.072	57.79 ± 12.285	−0.957	0.342
High blood pressure and/or diabetes mellitus			1.420	0.233
Yes	6	27		
No	11	25		
Lung disease			0.015	0.903
Yes	4	13		
No	13	39		
Weight (kg)	66.312 ± 11.044	70.154 ± 11.511	−1.206	0.232
BMI (kg/m^2^)	23.848 ± 3.880	25.577 ± 3.767	−1.631	0.108
ASA grading			0.585	0.444
1∼2	9 (29.0)	22 (71.0)		
3∼4	8 (21.1)	30 (78.9)		
Preoperative serum albumin (g/L)	43.935 ± 4.107	44.067 ± 4.167	−0.114	0.910
Duration of surgery (min)	125.82 ± 54.31	126.85 ± 31.26	−0.096	0.924
Intraoperative blood loss (mL)	20 (10, 35)	20 (10, 20)	0.518	0.605

**Table 6:** Results of multivariate logistic analysis affecting the prolongation of postoperative gastrointestinal function recovery time.

Variable	*β* value	OR value	95% CI	*P* value
Sarcopenia	2.654	14.207	(2.586, 78.041)	0.002
Age (years)	−0.085	0.556	(0.123, 2.515)	0.021
BMI (kg/m^2^)	−0.095	0.909	(0.754, 1.096)	0.319
Preoperative serum albumin (g/L)	−0.100	0.904	(0.752, 1.087)	0.285

## Discussion

The impact of preoperative sarcopenia on postoperative complications following fundoplication represents a clinically significant area warranting further investigation. Meanwhile, the increasing prevalence of HH concurrent with GERD has established laparoscopic anti-reflux surgery as one of the cornerstone interventions for this condition. Extensive research continues to validate its therapeutic efficacy and complication profiles, renewing clinical focus on modifiable risk factors (21).

LRHH combined with fundoplication [Nissen, Toupet or Dor (22–24)] primarily aims to reconstruct the anti-reflux barrier against gastric acid regurgitation. A retrospective analysis of 276 patients demonstrated no significant differences among the three fundoplication techniques in operative duration, intraoperative blood loss, hospital stay, or overall complication rates (25). During long-term follow-up, surgically treated patients exhibited superior outcomes in quality-of-life metrics, reflux symptom control, and medication independence compared to pharmacotherapy-only cohorts. The minimally invasive nature of laparoscopic anti-reflux surgery, characterized by reduced trauma and accelerated recovery, has cemented its status as the gold-standard approach.

While surgeon experience significantly influences complication profiles—with skilled operators achieving lower technical complication rates through refined techniques (26)—specific postoperative challenges persist. Notably, hypoalbuminemia, delayed gastrointestinal recovery, and dysphagia remain clinically relevant adverse events (3). These complications may be strongly associated with baseline nutritional status, highlighting sarcopenia as a potentially modifiable risk factor warranting preoperative optimization.

Sarcopenia, a prevalent nutritional-metabolic disorder in geriatric populations, is characterized by progressive loss of skeletal muscle mass and function. This condition manifests pathophysiological alterations including diminished protein synthetic capacity, dysregulated energy metabolism, and compromised immune competence (27). Furthermore, substantial evidence confirms that sarcopenia not only impairs ambulatory capacity and quality of life but also independently predicts postoperative complication risk across surgical specialties (28).

Our findings demonstrate that preoperative sarcopenia significantly correlates with the development of postoperative hypoalbuminemia and delayed gastrointestinal functional recovery—results aligning with established literature linking nutritional status to surgical complications (29). Mechanistically, sarcopenia-induced muscular atrophy and neuromuscular dysfunction may impair gastrointestinal motility, thereby prolonging postoperative enteric recovery (30). Critically, sarcopenia represents an independent risk factor for anti-reflux surgery complications. Consequently, preoperative identification of sarcopenic patients coupled with targeted nutritional optimization may enhance recovery trajectories and mitigate complication risks.

The impact of preoperative sarcopenia on postoperative complications and long-term outcomes following laparoscopic anti-reflux surgery constitutes a ​multifactorial interplay of diverse pathophysiological mechanisms. Our findings demonstrate that sarcopenia significantly correlates with postoperative hypoalbuminemia and delayed gastrointestinal recovery, providing novel insights into complication pathogenesis while establishing a ​conceptual framework for preoperative nutritional assessment and intervention. The observed decline in albumin is clinically relevant as it correlates with impaired wound healing and infection risk, underscoring the need for nutritional optimization in sarcopenic patients. Future research should focus on developing multimodal strategies—such as optimized nutritional support and structured resistance training—to improve perioperative care in patients with HH and GERD, thereby enhancing surgical outcomes and long-term quality of life. Furthermore, the routine implementation of preoperative sarcopenia screening and evidence-based interventions is strongly recommended to mitigate complication risks and facilitate comprehensive recovery.

This study has several limitations. First, its retrospective design may introduce unmeasured confounders despite multivariate adjustments. Second, the sample size is modest, which limits subgroup analyses and generalizability. Finally, we used CT-based muscle mass without functional assessments (e.g., grip strength), which may underdiagnose sarcopenia according to EWGSOP criteria. Future prospective studies with larger cohorts and functional measures are needed to validate our findings.

## Conclusion

Our findings show that while preoperative sarcopenia does not increase the risk of postoperative dysphagia, it significantly delays gastrointestinal function recovery and leads to lower postoperative albumin levels in GERD patients. This highlights the clinical importance of recognizing sarcopenia as a modifiable preoperative risk factor. We recommend routine sarcopenia screening in the preoperative assessment of GERD patients. For those diagnosed, a targeted prehabilitation program focusing on nutrition and physical training may improve surgical tolerance and recovery. Further studies are needed to validate the effectiveness of such prehabilitation and clarify the mechanisms by which sarcopenia impairs postoperative recovery.

## Data Availability

The original contributions presented in the study are included in the article/Supplementary Material, further inquiries can be directed to the corresponding author.

## References

[1] Abudureyimu K Maisiyiti A Azhatijiang Cheng Z Pildivas. Clinical efficacy study of laparoscopic hiatal hernia repair combined with anti-reflux surgery (report of 835 cases). Chin J Pract Surg. (2015) 35(11):1212–4. 10.7504/CJPS.ISSN1005-2208.2015.11.15

[2] Stefanidis D Hope WW Kohn GP Reardon PR Richardson WS Fanelli RD. SAGES Guidelines committee. Guidelines for surgical treatment of gastroesophageal reflux disease. Surg Endosc. (2010) 24(11):2647–69. 10.1007/s00464-010-1267-8 20725747

[3] Yadlapati R Hungness ES Pandolfino JE. Complications of antireflux surgery. Am J Gastroenterol. (2018) 113(8):1137–47. 10.1038/s41395-018-0115-7 29899438 PMC6394217

[4] Liu DS Wong DJ Goh SK Fayed A Stevens S Aly A PROTECTing antireflux surgery study group. Quantifying perioperative risks for antireflux and hiatus hernia surgery: a multicenter cohort study of 4301 patients. Ann Surg. (2024) 279(5):796–807. 10.1097/SLA.0000000000006223 38318704

[5] Ljungdalh JS Rubin KH Durup J Houlind KC. Reoperation after antireflux surgery: a population-based cohort study. Br J Surg. (2020) 107(12):1633–9. 10.1002/bjs.11672 32484246

[6] Rieger NA Jamieson GG Britten-Jones R Tew S. Reoperation after failed antireflux surgery. Br J Surg. (1994) 81(8):1159–61. 10.1002/bjs.1800810825 7953347

[7] Iwaki M Kobayashi T Nogami A Saito S Nakajima A Yoneda M. Impact of sarcopenia on non-alcoholic fatty liver disease. Nutrients. (2023) 15(4):891. 10.3390/nu15040891 36839249 PMC9965462

[8] Cruz-Jentoft AJ Baeyens JP Bauer JM Boirie Y Cederholm T Landi F European Working group on sarcopenia in older people. Sarcopenia: european consensus on definition and diagnosis: report of the European working group on sarcopenia in older people. Age Ageing. (2010) 39(4):412–23. 10.1093/ageing/afq034 20392703 PMC2886201

[9] Cruz-Jentoft AJ Bahat G Bauer J Boirie Y Bruyère O Cederholm T Writing group for the European working group on sarcopenia in older people 2 (EWGSOP2), and the extended group for EWGSOP2. Sarcopenia: revised European consensus on definition and diagnosis. Age Ageing. (2019) 48(4):601. 10.1093/ageing/afz046 Erratum for: Age Ageing. 2019 January 1;48(1):16-31. 31081853 PMC6593317

[10] Zanker J Sim M Anderson K Balogun S Brennan-Olsen SL Dent E Consensus guidelines for sarcopenia prevention, diagnosis and management in Australia and New Zealand. J Cachexia Sarcopenia Muscle. (2023) 14(1):142–56. 10.1002/jcsm.13115 36349684 PMC9891980

[11] Park B Bhat S Xia W Barazanchi AWH Frampton C Hill AG Consensus-defined sarcopenia predicts adverse outcomes after elective abdominal surgery: meta-analysis. BJS Open. (2023) 7(4):zrad065. 10.1093/bjsopen/zrad065 37542472 PMC10404004

[12] Baglietto N Vaquero-Cristóbal R Albaladejo-Saura M Mecherques-Carini M Esparza-Ros F. Assessing skeletal muscle mass and lean body mass: an analysis of the agreement among dual x-ray absorptiometry, anthropometry, and bioelectrical impedance. Front Nutr. (2024) 11:1445892. 10.3389/fnut.2024.1445892 39224178 PMC11366593

[13] He Q Xia W. Analysis of the current status of computed tomography diagnosis of sarcopenia. Arch Med Sci. (2024) 21(2):374–82. 10.5114/aoms/191297 40395884 PMC12087324

[14] Lavalle S Scapaticci R Masiello E Messina C Aliprandi A Mario Salerno V Advancements in sarcopenia diagnosis: from imaging techniques to non-radiation assessments. Front Med Technol. (2024) 6:1467155. 10.3389/fmedt.2024.1467155 39445171 PMC11496100

[15] Ebadi M Bhanji RA Mazurak VC Montano-Loza AJ. Sarcopenia in cirrhosis: from pathogenesis to interventions. J Gastroenterol. (2019) 54(10):845–59. 10.1007/s00535-019-01605-6 31392488 PMC6759678

[16] Chen LK Woo J Assantachai P Auyeung TW Chou MY Iijima K Asian working group for sarcopenia: 2019 consensus update on sarcopenia diagnosis and treatment. J Am Med Dir Assoc. (2020) 21(3):300–307.e2. 10.1016/j.jamda.2019.12.012 32033882

[17] Chan MY Chok KSH. Sarcopenia in pancreatic cancer - effects on surgical outcomes and chemotherapy. World J Gastrointest Oncol. (2019) 11(7):527–37. 10.4251/wjgo.v11.i7.527 31367272 PMC6657219

[18] Lin Z Li Y Wu J Zheng H Yang C. Nomogram for prediction of prolonged postoperative ileus after colorectal resection. BMC Cancer. (2022) 22(1):1273. 10.1186/s12885-022-10377-x 36474177 PMC9724353

[19] Taicheng Z Zhiwei H Yiliang L Enmin H Ning M Zehui H Standardized seven step laparoscopic surgery guide for hiatal hernia and gastroesophageal reflux disease (2025 edition). Chin J Gen Surg. (2025) 34(04):600–13. 10.7659/j.issn.1005-6947.250074

[20] Granderath FA. Measurement of the esophageal hiatus by calculation of the hiatal surface area (HSA). why, when and how? Surg Endosc. (2007) 21(12):2224–5. 10.1007/s00464-007-9348-z 17479323

[21] Bonavina L Bona D Aiolfi A Shabat G Annese V Galassi L. Fundoplication: old concept for novel challenges? Visc Med. (2024) 40(5):236–41. 10.1159/000536566 39398391 PMC11466449

[22] Csendes A. Laparoscopic nissen fundoplication. Surgery. (2018) 164(5):1126–34. 10.1016/j.surg.2018.03.003 29807647

[23] Wenck C Zornig C. Laparoscopic toupet fundoplication. Langenbecks Arch Surg. (2010) 395(4):459–61. 10.1007/s00423-010-0637-y 20354723

[24] Frazzoni M Piccoli M Conigliaro R Frazzoni L Melotti G. Laparoscopic fundoplication for gastroesophageal reflux disease. World J Gastroenterol. (2014) 20(39):14272–9. 10.3748/wjg.v20.i39.14272 25339814 PMC4202356

[25] Yanchun P Xiangyao L Siwei Z. Clinical comparison of laparoscopic repair of hiatal hernia combined with different anti reflux techniques for the treatment of hiatal hernia complicated with gastroesophageal reflux disease. Chin J Endosc. (2019) 25(04):11–8. 10.3969/j.issn.1007-1989.2019.04.003

[26] Hunter JG Swanstrom L Waring JP. Dysphagia after laparoscopic antireflux surgery. The impact of operative technique. Ann Surg. (1996) 224(1):51–7. 10.1097/00000658-199607000-00008 8678618 PMC1235246

[27] Sayer AA Cruz-Jentoft A. Sarcopenia definition, diagnosis and treatment: consensus is growing. Age Ageing. (2022) 51(10):afac220. 10.1093/ageing/afac220 36273495 PMC9588427

[28] Jones K Gordon-Weeks A Coleman C Silva M. Radiologically determined sarcopenia predicts morbidity and mortality following abdominal surgery: a systematic review and meta-analysis. World J Surg. (2017) 41(9):2266–79. 10.1007/s00268-017-3999-2 28386715 PMC5544798

[29] Hu WH Cajas-Monson LC Eisenstein S Parry L Cosman B Ramamoorthy S. Preoperative malnutrition assessments as predictors of postoperative mortality and morbidity in colorectal cancer: an analysis of ACS-NSQIP. Nutr J. (2015) 14:91. 10.1186/s12937-015-0081-5 26345703 PMC4561437

[30] Wagner D DeMarco MM Amini N Buttner S Segev D Gani F Role of frailty and sarcopenia in predicting outcomes among patients undergoing gastrointestinal surgery. World J Gastrointest Surg. (2016) 8(1):27–40. 10.4240/wjgs.v8.i1.27 26843911 PMC4724585

